# The Effect of Mock Code Blue Simulations and Dedicated Advanced Cardiac Life Support Didactics on Resident Perceived Competency

**DOI:** 10.7759/cureus.11705

**Published:** 2020-11-25

**Authors:** Dallis Q Ngo, Christina Vu, Thien Nguyen, Patricia Sotolongo, Manika Talati, Nikki Zahabi, Katrina Platt

**Affiliations:** 1 Pulmonary Medicine, Saint Peter’s University Hospital, New Brunswick, USA; 2 Infectious Diseases, University of Southern California, Los Angeles, USA; 3 Internal Medicine, Desert Regional Medical Center, Palm Springs, USA; 4 Internal Medicine, Scripps Clinic, Oceanside, USA; 5 Internal Medicine, Hoag Medical Group, Newport Beach, USA

**Keywords:** pulmonary critical care, medical residency, internal medicine, critical care, quality improvement research, simulation medicine, cardiac resuscitation, code blue

## Abstract

In-hospital cardiac or pulmonary arrest is associated with high mortality. In the USA, approximately 200,000 of these events occur and are associated with overall survival rates of 18%-20%. Despite advances in resuscitative methods, the probability of intact survival also remains unfavorable. Though many factors play a role, we believe a large portion of a patient’s survival is dependent on the competency of the leader of the code blue or resuscitative team’s efforts. Newly minted physicians who enter medical training in their respective residencies are equipped with a wide range of clinical competency in regards to hands-on experience and aptitude with handling code blue scenarios. Through the use of mock code blue simulations along with dedicated didactics over a seven-month time span, we were able to demonstrate success in improving clinical competency and patient survival outcomes.

## Introduction

The rapid sequence of events during a code blue scenario, most often as a result of a cardiac or pulmonary arrest, can be a frightening experience for everyone, including the patient, their family, and even the medical team running the code. But despite the advances in resuscitation methodologies over the past decade, the probability of intact survival from in-hospital cardiac arrest remains unfavorable. In 2009, the rate of clinically significant neurological disability among survivors was reported to occur in 28.1%-37.7% cases [1-2]. Successful outcomes occur as a result of immediate and coordinated actions carried out by the healthcare providers. One potential barrier to success lies within the variations in the level of experience providers are equipped with when dealing with resuscitative efforts during a code blue scenario [3].

We believe that an effective code blue team must have effective communication skills, clear roles and responsibilities among members, as well as the medical proficiency necessary to carry out their individual responsibilities. Furthermore, we believe that the lead physician’s role during resuscitative efforts is to ensure that the team operates efficiently and safely. This implies that the physician should be proficient with Advanced Cardiac Life Support (ACLS) algorithms, performing emergent procedures, knowing how to operate basic equipment found in standard crash carts, making accurate medical assessments of underlying medical derangements, and carrying out appropriate interventions.

Despite Basic Life Support (BLS) and ACLS certification as required prior to starting training in residency training programs in the USA, this gap in clinical competency with regards to a resident’s ability to safely and comfortably take a leadership role in running a code blue was demonstrated in a questionnaire survey at the start of the academic year.

In order to accomplish and maintain this level of competency for future physicians, we believe that resident physicians should have more exposure to emergent response lectures and simulations as part of their regular didactic schedule.

Purpose

To ensure the safety of patients and the medical team during a code blue event, we believe that code blue or emergent response lectures and simulations should be implemented or expanded upon for resident physicians worldwide. Currently, the majority of resident physicians have minimal exposure to ACLS or code blue training outside of BLS/ACLS certification courses. Another challenge to competency in resuscitation is the infrequency of events. In the USA, in-hospital cardiac arrest occurs approximately 200,000 times a year, with an incidence rate of 4.5 cases per 1,000 hospitalizations [4]. This means that there is often a considerable lapse in time between hands-on experience during actual in-hospital events. With too little or too infrequent exposure and the practice of emergent situations, medical residents are deprived of valuable opportunities to learn various skills in medicine, procedures, and leadership.

This project aims to determine if the ACLS emergency response training and simulations for internal medicine residents of Desert Regional Medical Center will result in a self-reported increased clinical competency and comfort in taking a leadership position during a code blue with the ultimate goal of improving patient outcomes during cardiac resuscitation efforts demonstrated by quality metrics.

## Materials and methods

This study was performed at Desert Regional Medical Center (DRMC) in Palm Springs, California, over a seven-month period from October 2018 to May 2019. To establish a baseline and assess the Desert Regional Medical Center internal medicine residents’ competency and comfort in taking a leadership role during a code blue situation, as well as the perceived ability to operate necessary equipment and administer medications, a 12-question survey we developed was administered to all residents at each training level (Table 1). A total of 19 residents, from a cohort of 33, were recruited for the initial survey in October 2018. Once the results were obtained, simulated code blue scenarios were scheduled monthly throughout the academic year for all interns and residents of the internal medicine residency program. These simulated code blue scenarios were staffed with the appropriate hospital nursing staff, code blue crash cart, mannequins, and telemetry monitors as typically seen in a real-life event. Didactic lectures were provided to the internal medicine residents to provide additional knowledge and reinforcement regarding ACLS algorithms, electrocardiogram (ECG) rhythm recognition, and appropriate medication usage. An identical survey was administered at the conclusion of the study in May 2019 and results from 24 residents were obtained. Monthly quality metrics data from October 2018 through May 2019 at Desert Regional Medical Center were obtained from the Get With The Guidelines® Resuscitation database measuring the length of cardiac arrest, the reason cardiac resuscitation efforts ended, and patient hospital discharge status after cardiac resuscitation. Quality metrics data for May 2019 were omitted from analysis at the conclusion of the study secondary to an incomplete data set. This study was approved at our institution by the Institutional Review Board.

**Table 1:** Survey administered to internal medicine residents during the beginning of the study in October 2018 and at the end of the study in May 2019

	Strongly Disagree	Disagree	Agree	Strongly Agree
I am confident in my abilities and I am comfortable in running a code blue without an attending physician				
I have a clear understanding of my role during a Code Blue				
I feel comfortable announcing my role and communicating it with the resuscitation team during a crisis				
I feel comfortable assessing the effectiveness of chest compressions				
I feel comfortable drawing up resuscitation medications during a code blue scenario				
I feel comfortable operating the defibrillator during a code blue				
The use of mock code blue drills will play an important role in preparing me to handle crisis situations in the future				
Practicing multi-disciplinary, team-based mock code blue scenarios at DRMC would make me more comfortable in code blue situations in the future				
Practicing multi-disciplinary, team-based mock code blue scenarios will improve patient outcome				
Code blue and crisis management training should be mandatory for all residents at DRMC				
Team debriefing after a code blue or crisis scenario is important				
I would like to participate more in simulated crisis scenarios and mock codes and believe that they should be held more frequently				

## Results

**Figure 1:**
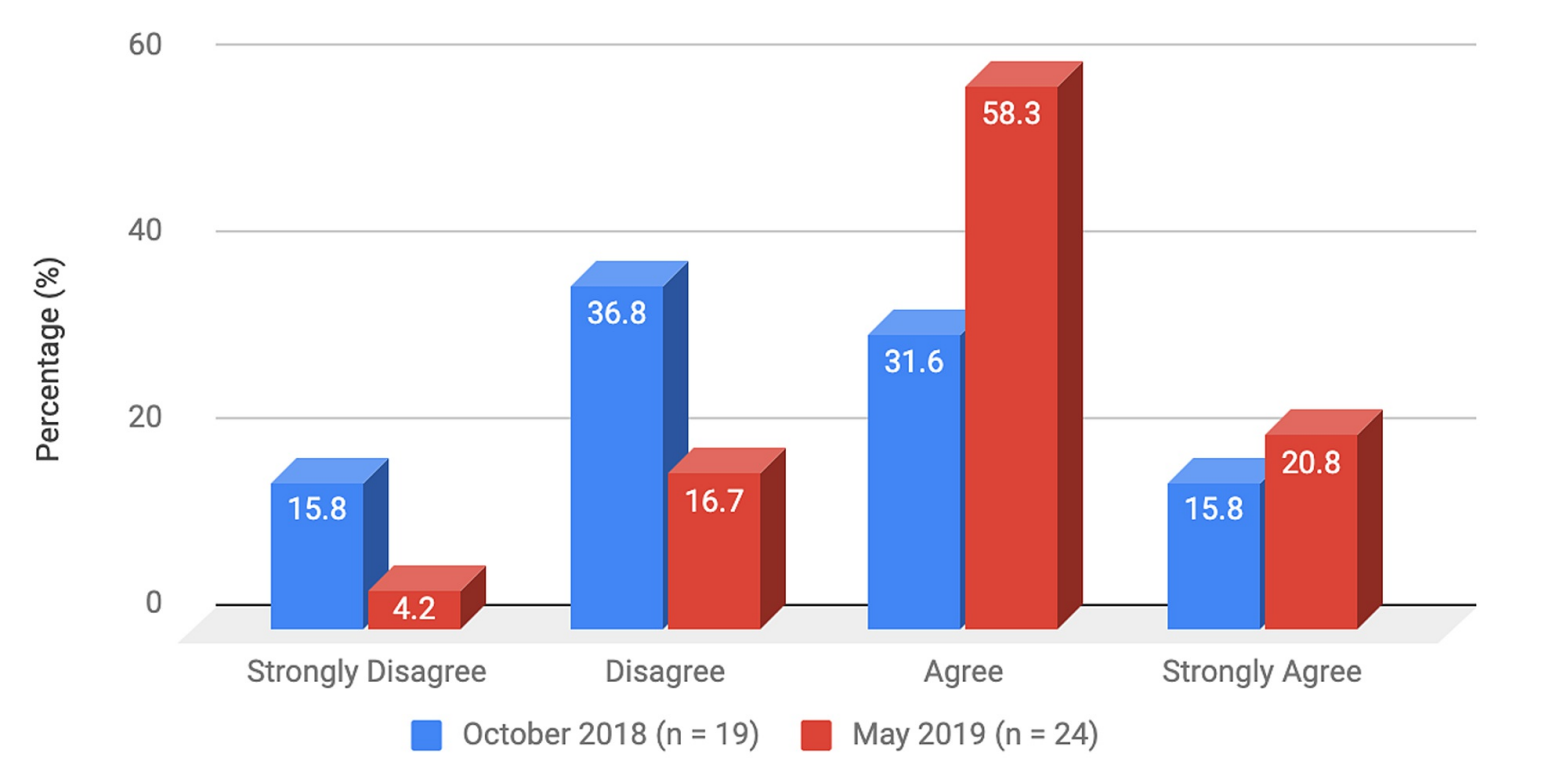
Survey question 1 I am confident in my abilities and I am comfortable in running a code without an attending physician

**Figure 2:**
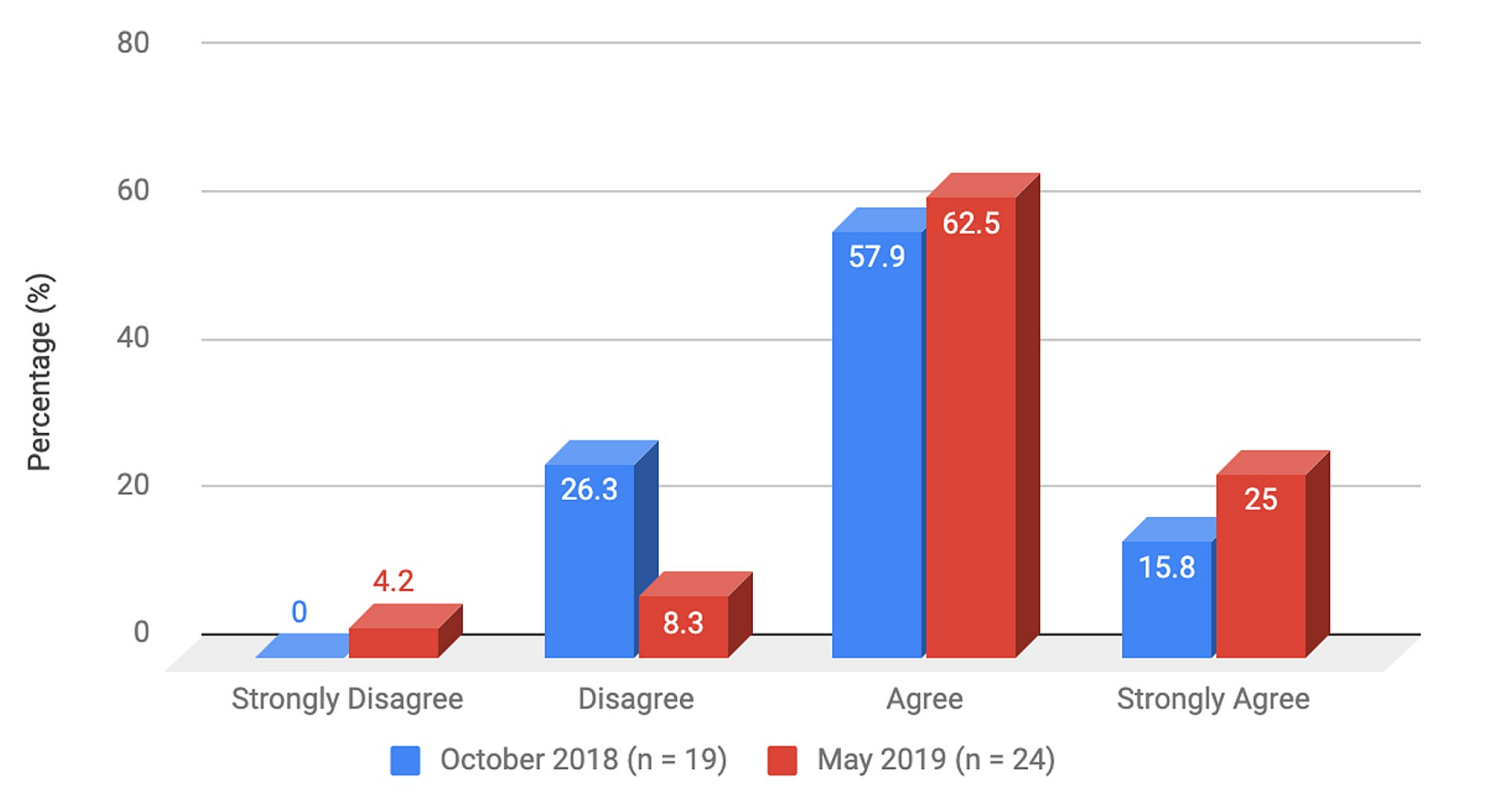
Survey question 2 I have a clear understanding of my role during a code blue

**Figure 3:**
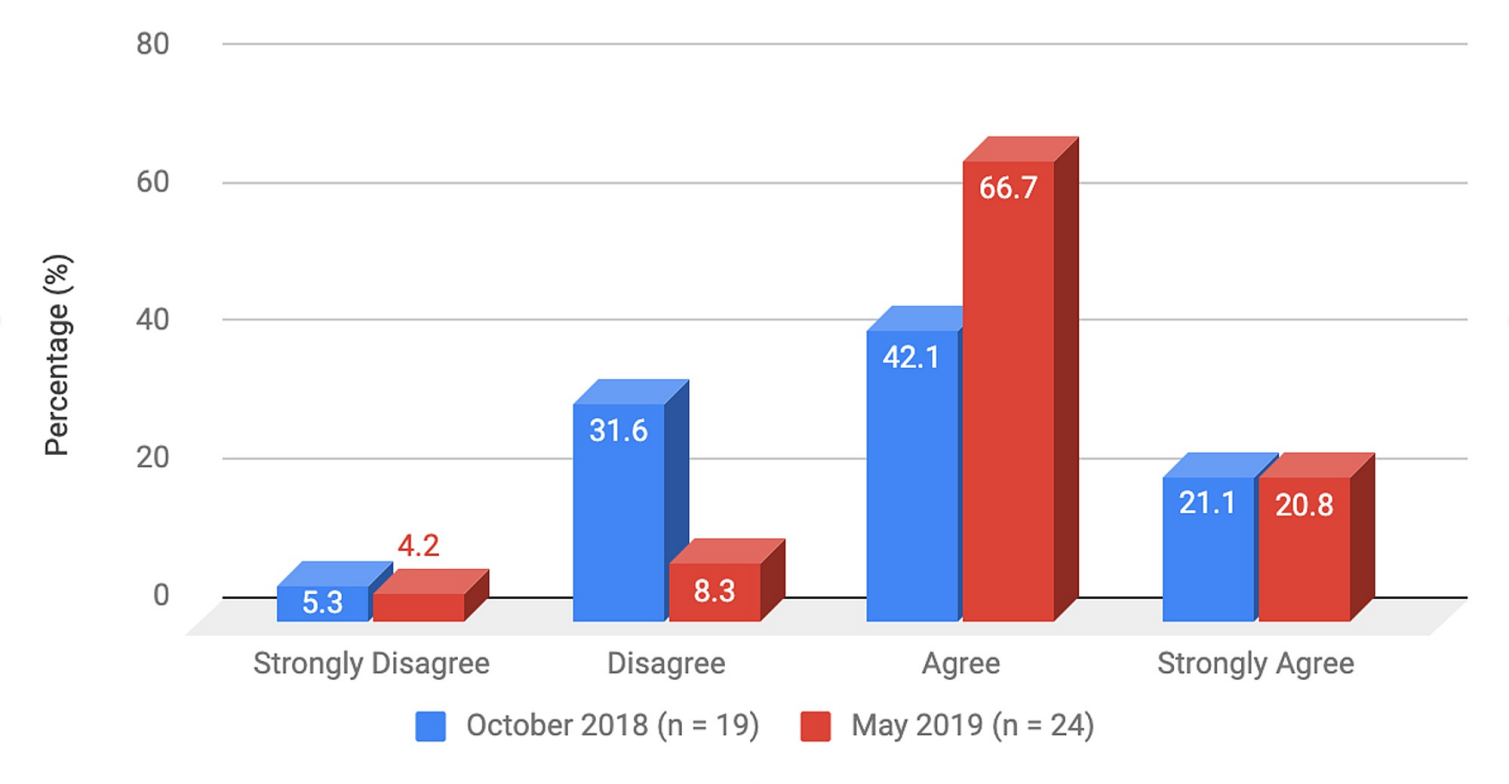
Survey question 3 I feel comfortable announcing my role and communicating it with the resuscitation team during a crisis

**Figure 4:**
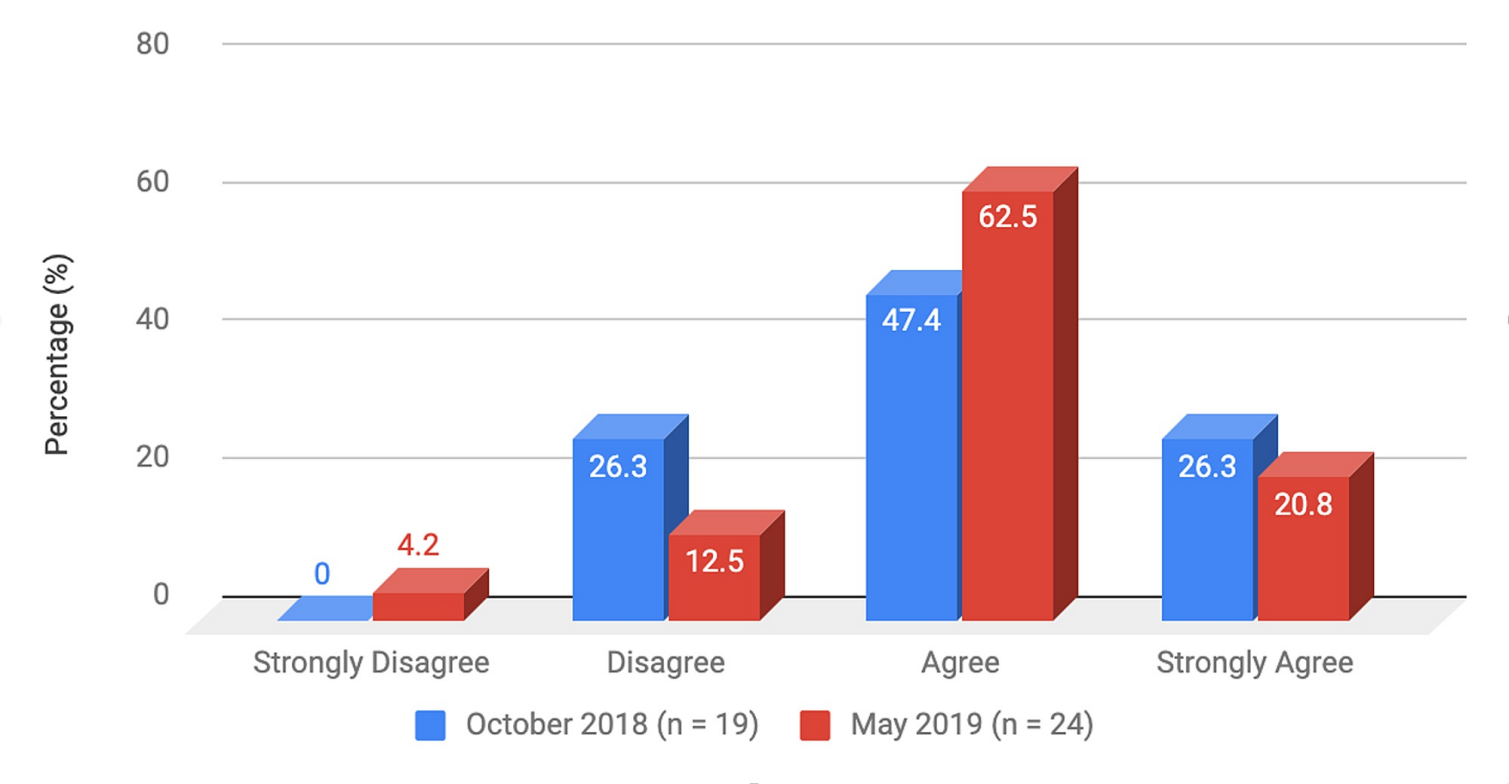
Survey question 4 I feel comfortable assessing the effectiveness of chest compressions

**Figure 5:**
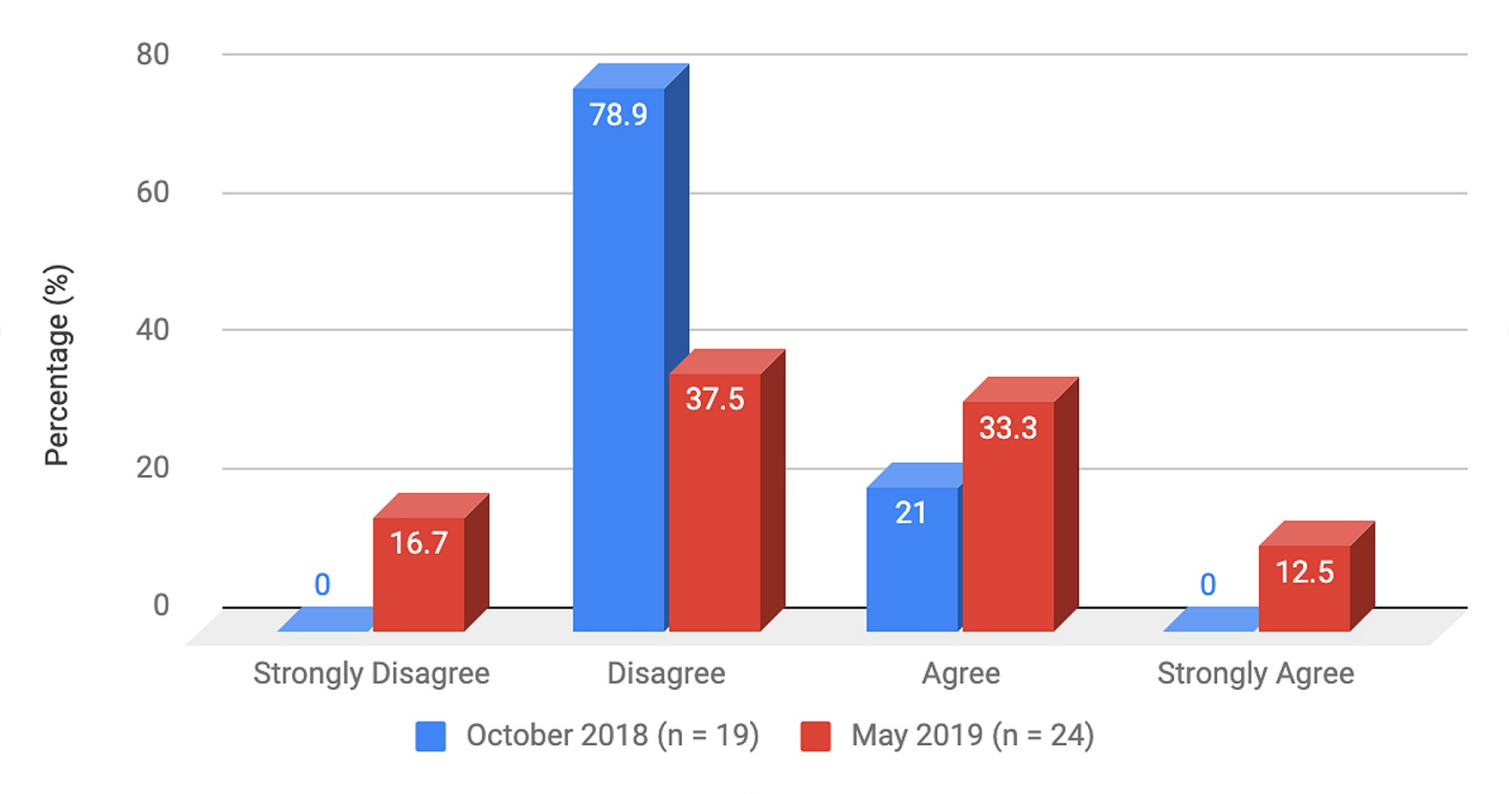
Survey question 5 I feel comfortable drawing up resuscitation medications during a code blue scenario

**Figure 6:**
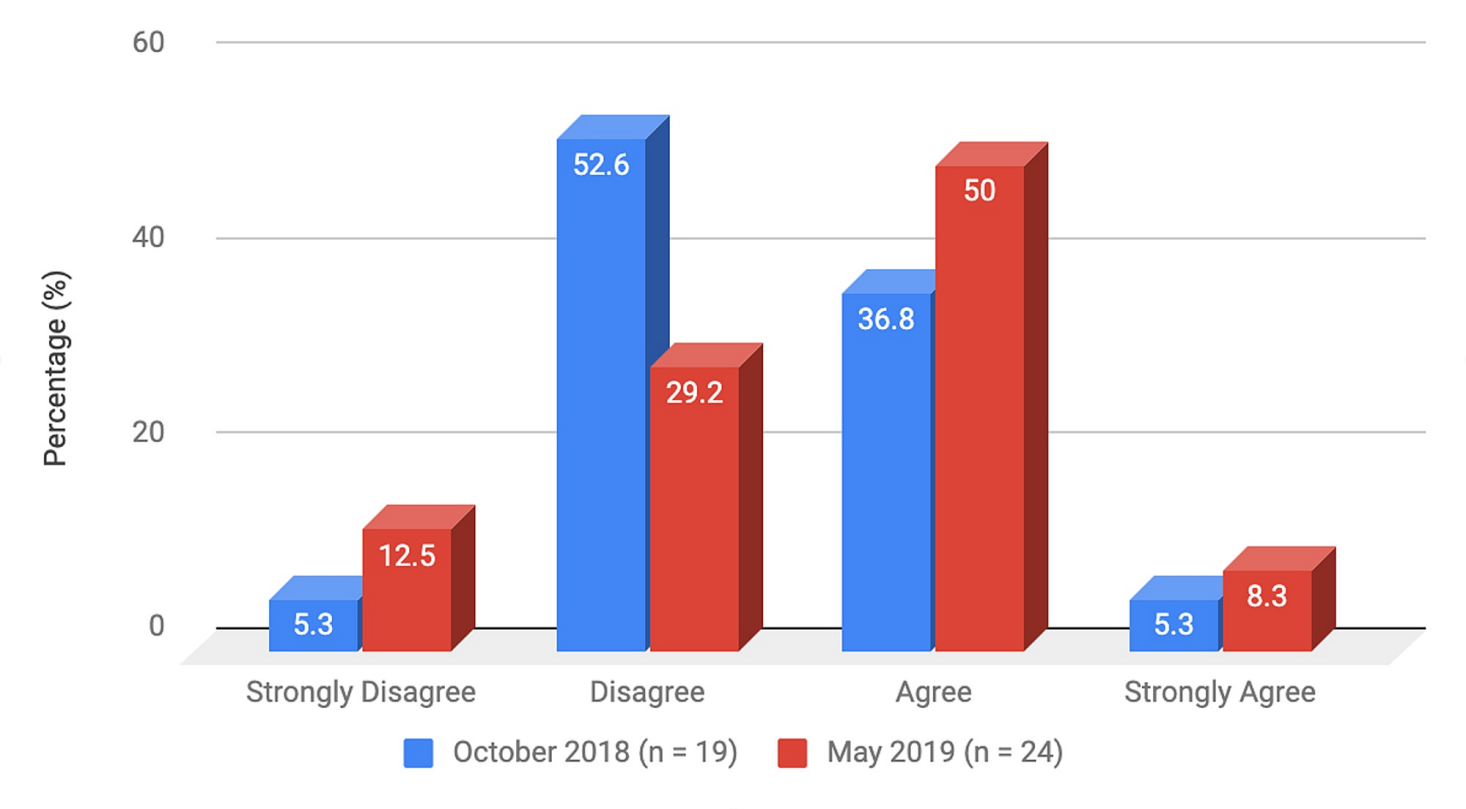
Survey question 6 I feel comfortable operating the defibrillator during a code blue

**Figure 7:**
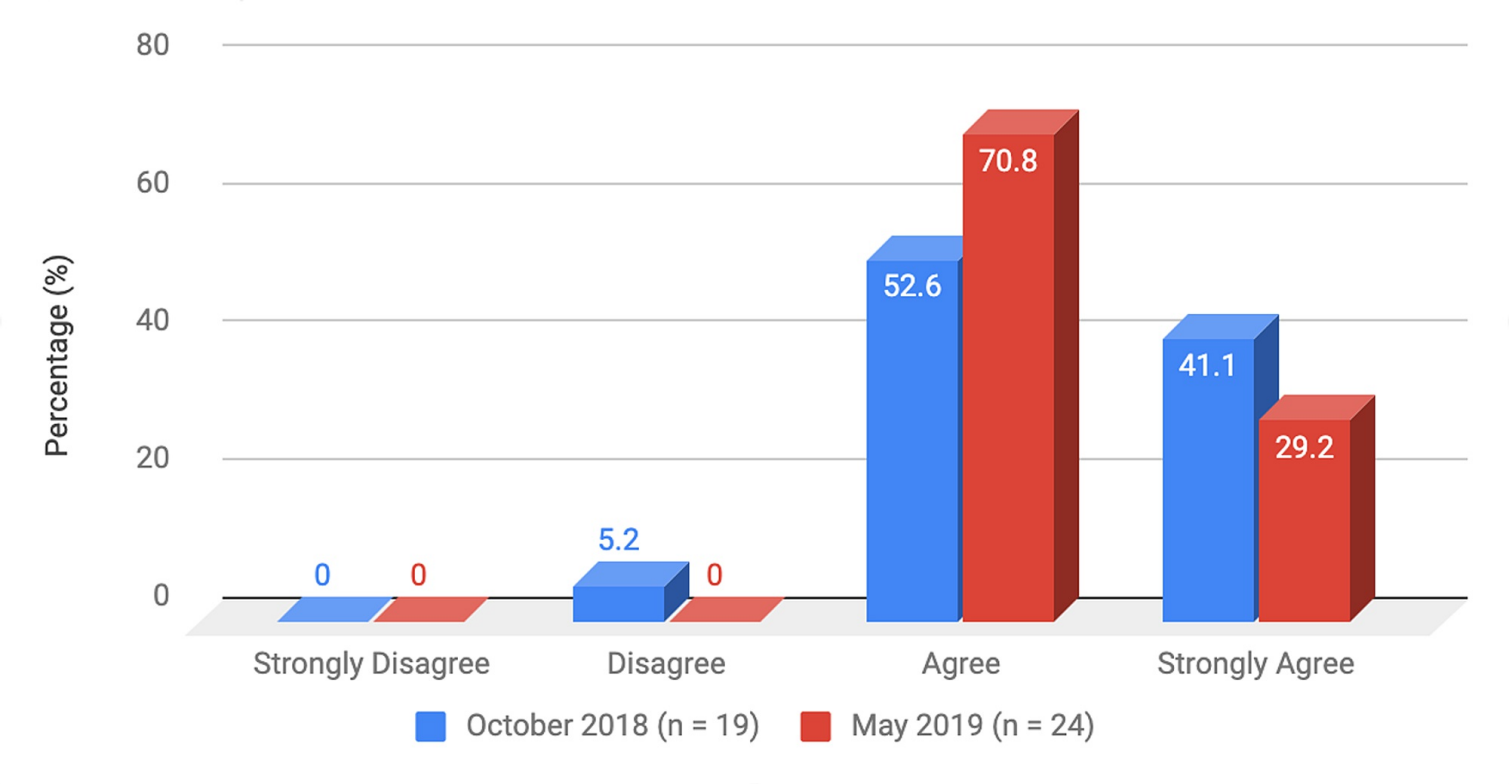
Survey question 7 The use of mock code blue drills will play an important role in preparing me to handle crisis situations in the future

**Figure 8:**
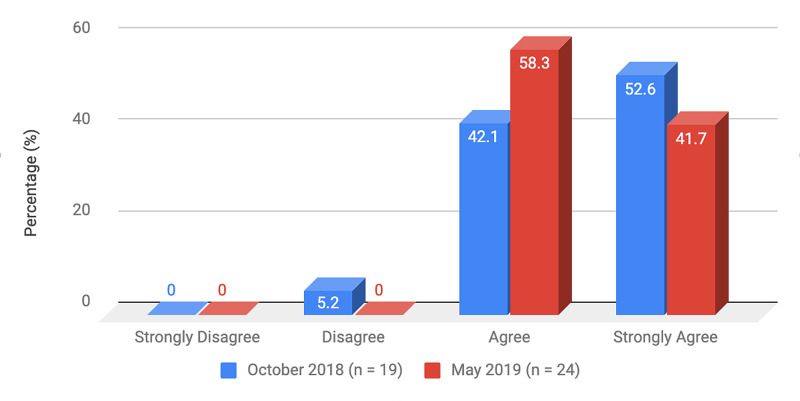
Survey question 8 Practicing multi-disciplinary, team-based mock code blue scenarios at DRMC would make me more comfortable in code blue situations in the future DRMC: Desert Regional Medical Center

**Figure 9:**
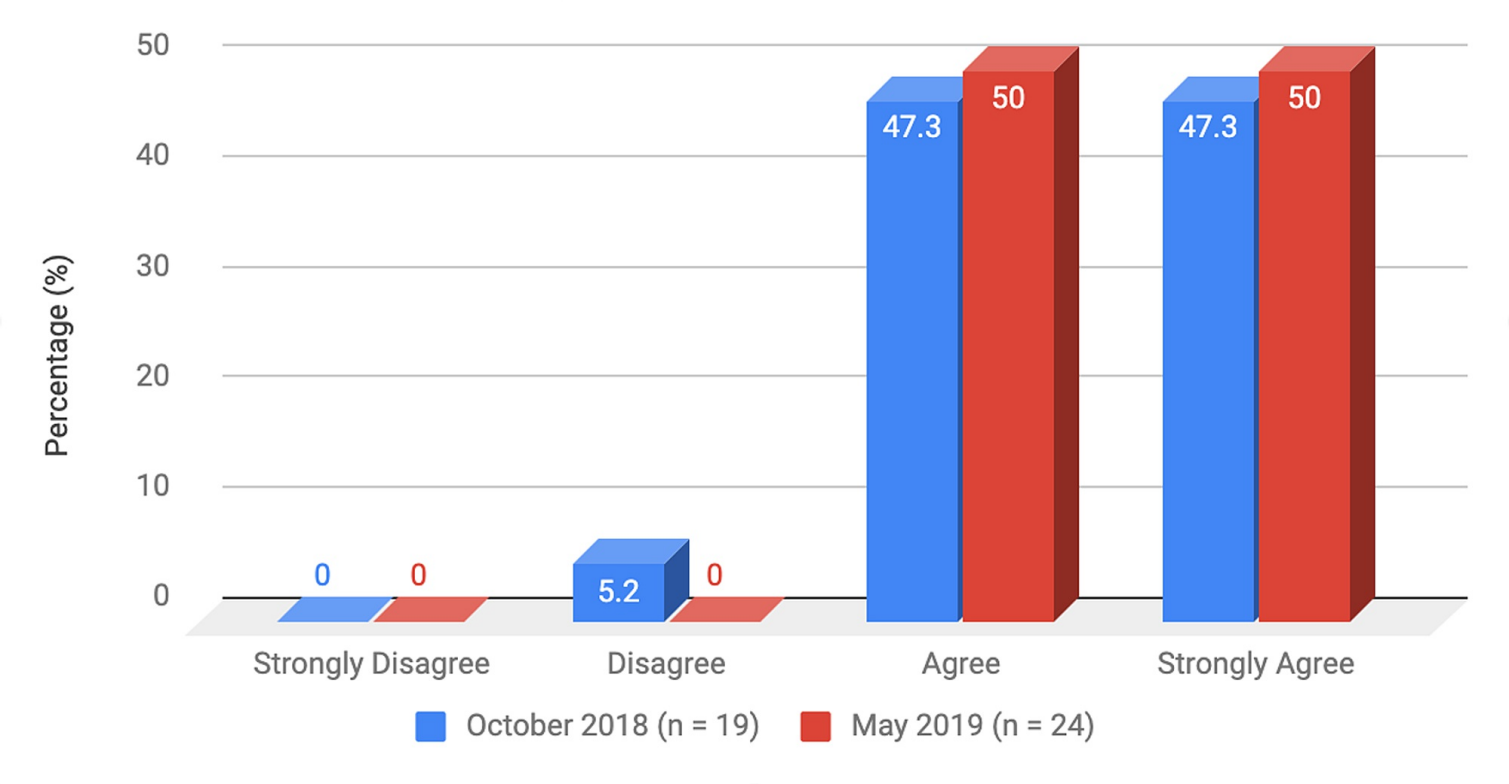
Survey question 9 Practicing multi-disciplinary, team-based mock code blue scenarios will improve patient outcome

**Figure 10:**
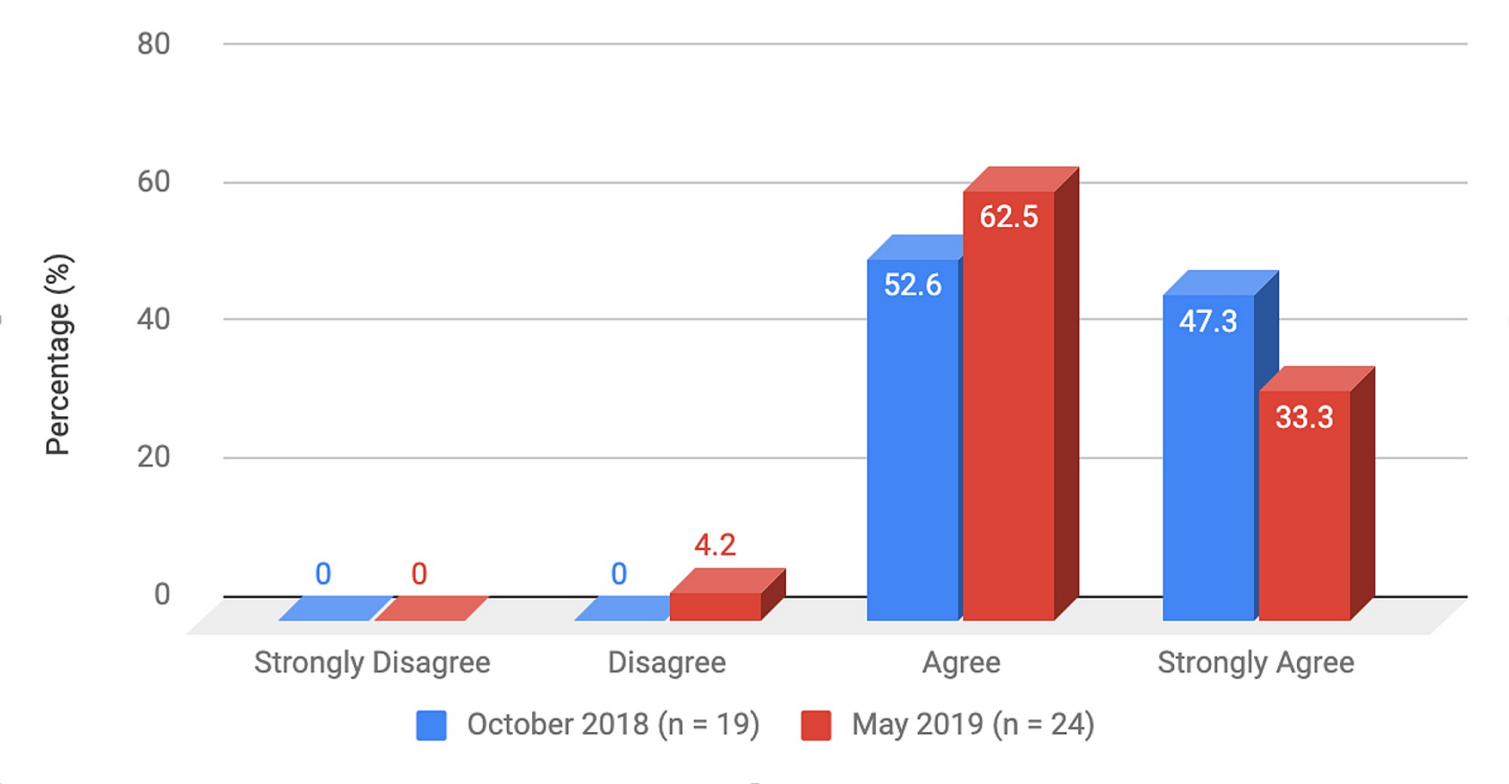
Survey question 10 Code blue and crisis management training should be mandatory for all residents at DRMC DRMC: Desert Regional Medical Center

**Figure 11:**
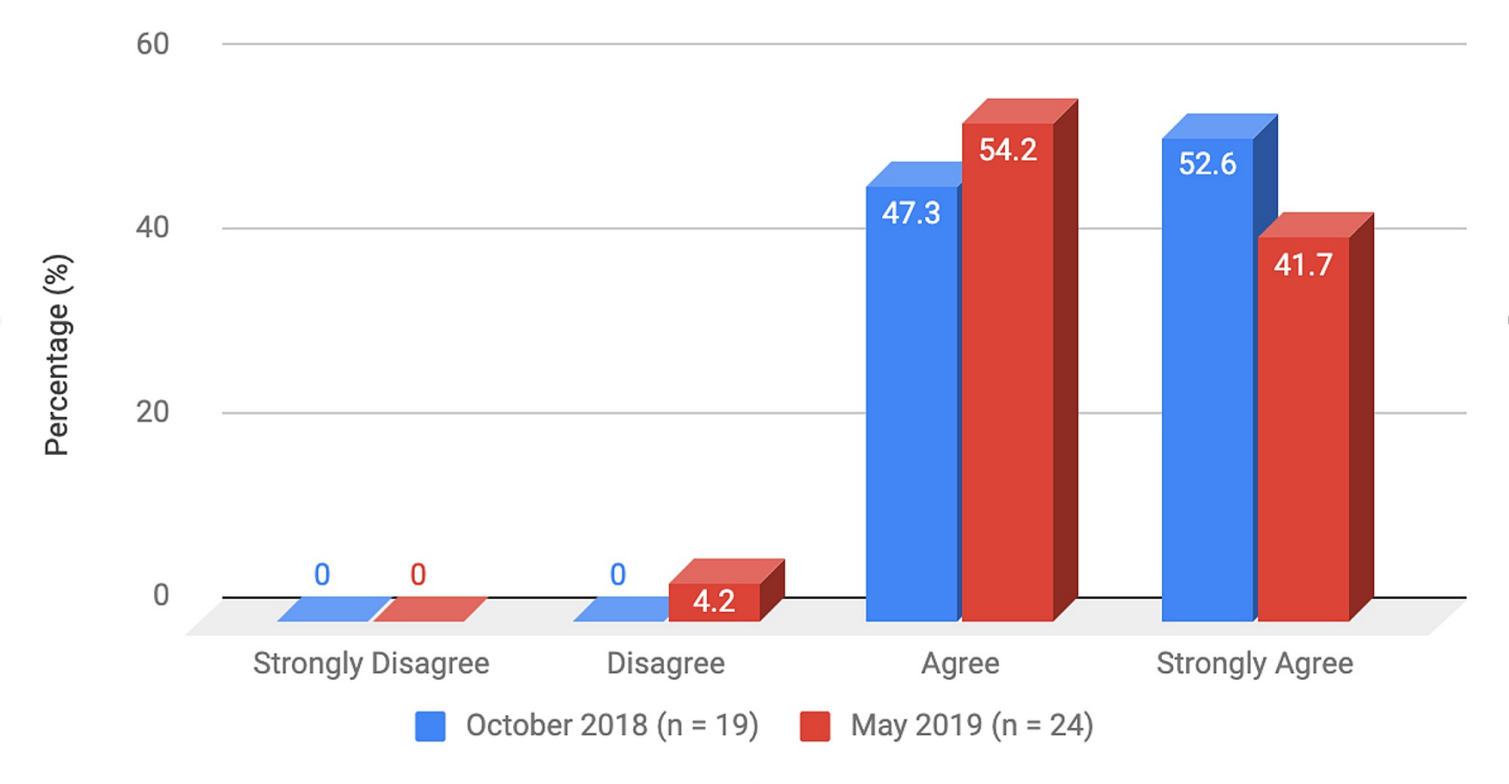
Survey question 11 Team debriefing after a code blue or crisis scenario is important

**Figure 12:**
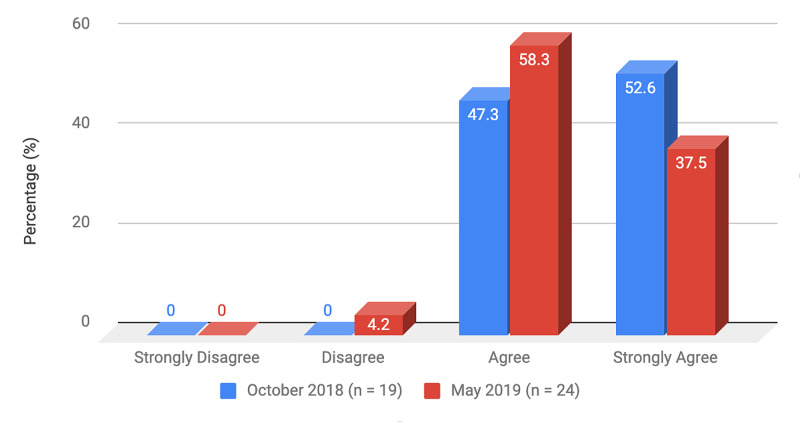
Survey question 12 I would like to participate more in simulated crisis scenarios and mock codes and believe that they should be held more frequently

**Figure 13:**
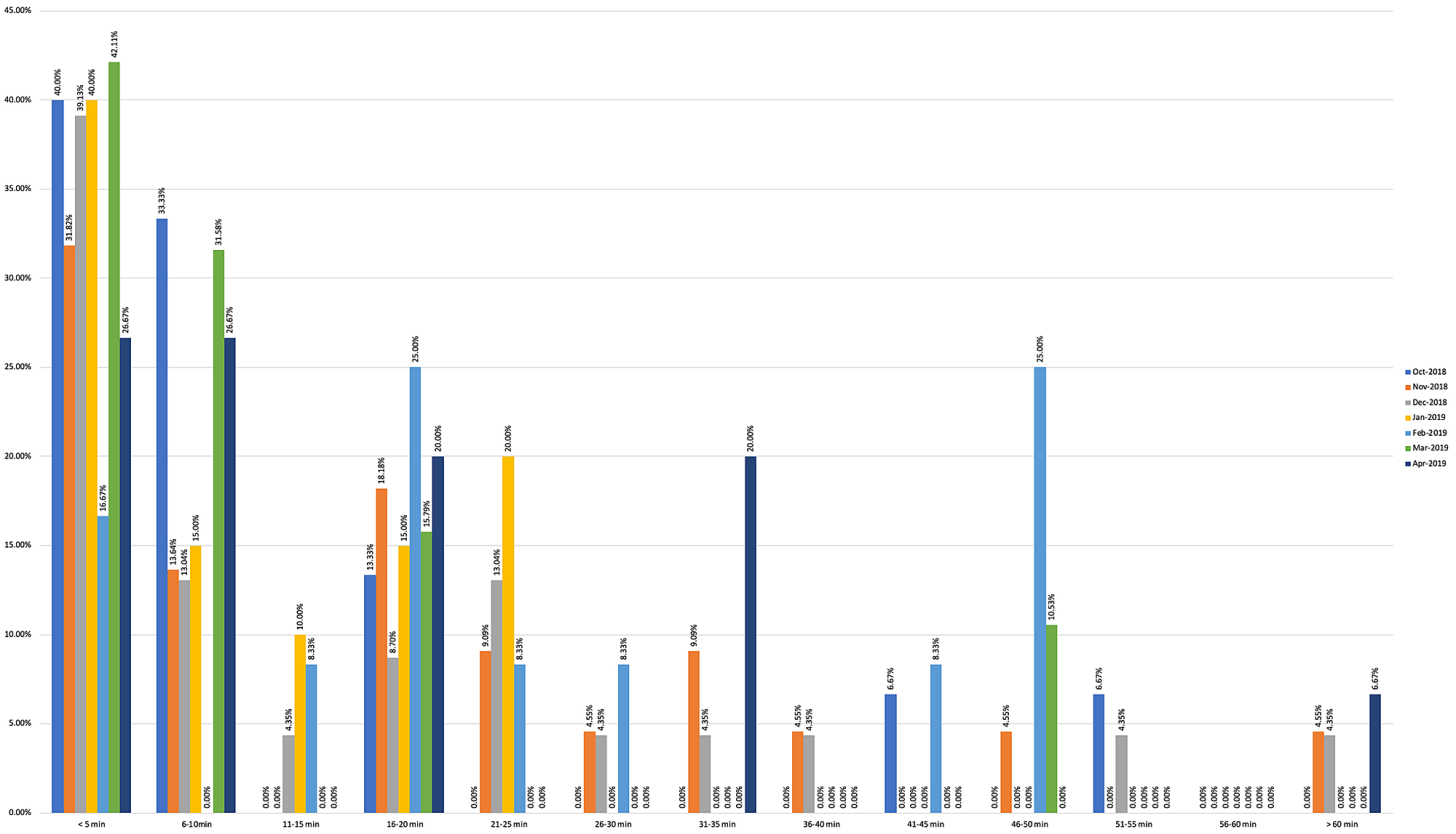
Length of resuscitation efforts

**Figure 14:**
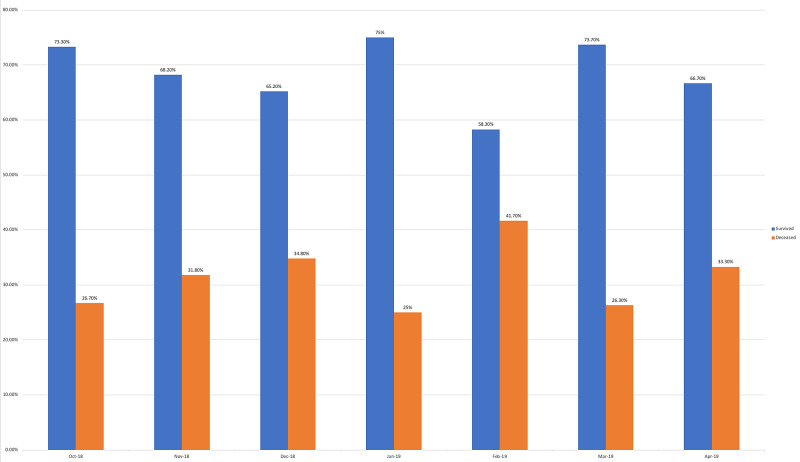
Reason resuscitation efforts ended

**Figure 15:**
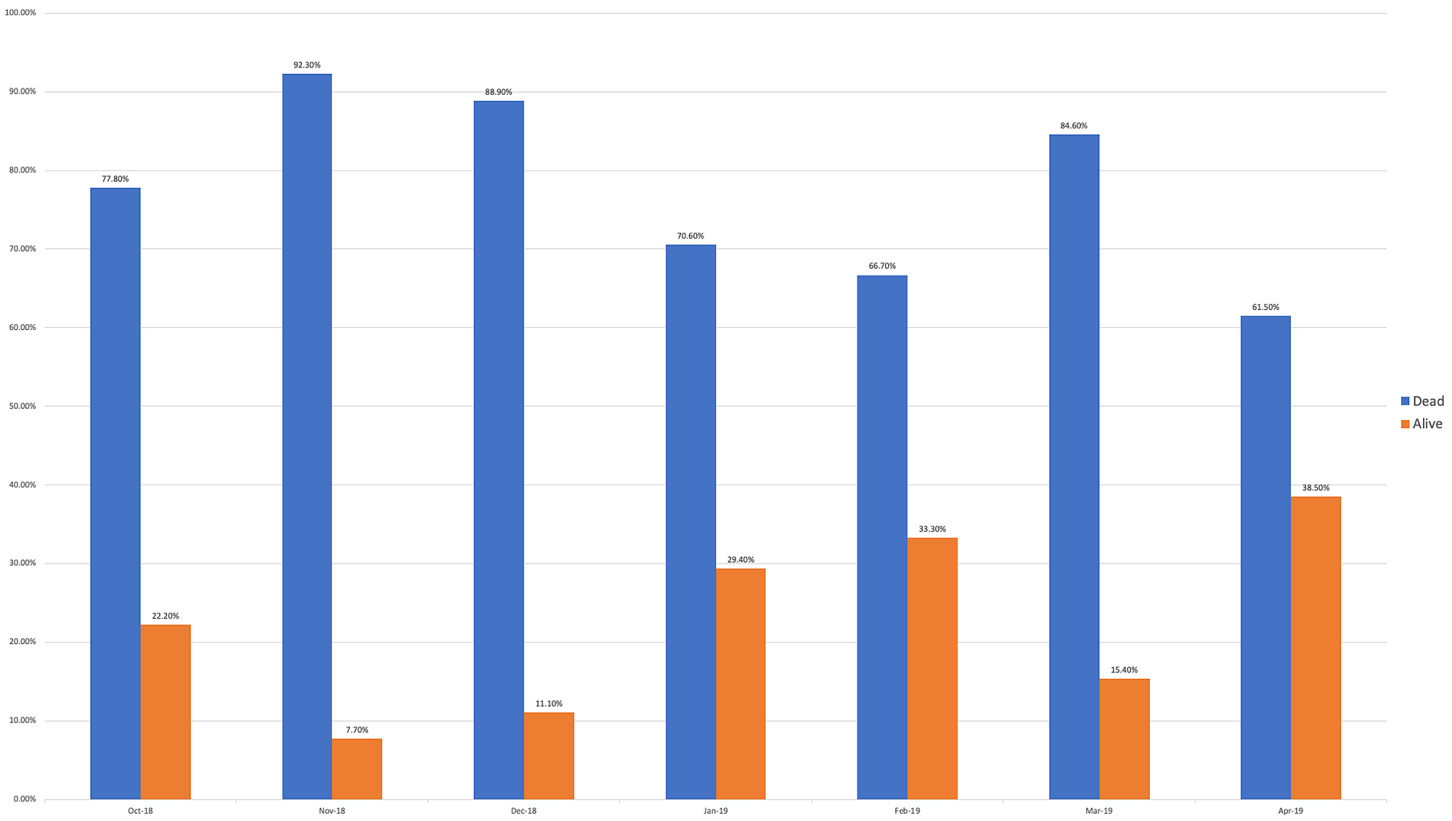
Hospital discharge status after cardiac arrest

Survey results from internal medicine residents at Desert Regional Medical Center at the beginning and end of the study are listed in Tables 2-3. At the end of the study period, there was an improvement from 31.6% to 58.3% and 15.8% to 20.8% in residents that agreed and strongly agreed, respectively, with regards to confidence in their ability to lead a code blue without an attending physician present (Figure 1). More residents had a clear understanding of their role during a code, an increase from 57.9% to 62% for individuals who agreed (Figure 2). There was a 24.6% increase in residents who felt more comfortable announcing their role and communicating it to the resuscitation team during a crisis (Figure 3). There was an increase from 47.4% to 62% of residents who felt comfortable assessing the effectiveness of chest compressions (Figure 4). With regards to the comfort level with drawing up resuscitation medications during a code blue scenario, there was a decrease in disagreement from 78.9% to 37.5% (Figure 5). There was a rise in the agreement of comfort with operating the defibrillator and the notion of belief that mock code blue drills will prepare them for the ability to handle crisis situations to 50% and 70.8%, respectively (Figures 6-7). Of the residents, 58.3% and 50% believe that practicing with multi-disciplinary teams during mock code blues will make them more comfortable in future code blue situations and will improve patient outcomes respectively (Figures 8-9). In addition, 62.5% of residents agree that code blue training should be mandatory for all residents at our institution; however, there was an increase from 0% to 4.2% of residents who disagree at the end of the study period (Figure 10). There was an increase from baseline in residents that agreed that team debriefing after a code blue is important and desire to participate in more simulated code blues at the end of the study (Figures 11-12). A majority of resuscitation efforts throughout the study had durations of five minutes or less (Figure 13). Throughout the study period, there was a rise in the percentage of deaths, as the reason resuscitation events ended as high as 41.7% (Figure 14); however, there was also a rise in the percentage of patients who were discharged alive at the end of their hospitalization (Figure 15).

**Table 2:** Survey questionnaire results at the start of the study DRMC: Desert Regional Medical Center

October 2018 (n = 19)	Strongly Disagree	Disagree	Agree	Strongly Agree
I am confident in my abilities and I am comfortable in running a code blue without an attending physician (%)	15.8	36.8	31.6	15.8
I have a clear understanding of my role during a code blue (%)	0	26.3	57.9	15.8
I feel comfortable announcing my role and communicating it with the resuscitation team during a crisis (%)	5.3	31.6	42.1	21.1
I feel comfortable assessing the effectiveness of chest compressions (%)	0	26.3	47.4	26.3
I feel comfortable drawing up resuscitation medications during a code blue scenario (%)	0	78.9	21.1	0
I feel comfortable operating the defibrillator during a code blue (%)	5.3	52.6	36.8	5.3
The use of mock code blue drills will play an important role in preparing me to handle crisis situations in the future (%)	0	5.2	52.6	42.1
Practicing multi-disciplinary, team-based mock code blue scenarios at DRMC would make me more comfortable in code blue situations in the future (%)	0	5.2	42.1	52.6
Practicing multi-disciplinary, team-based mock code blue scenarios will improve patient outcome (%)	0	5.2	47.3	47.3
Code blue and crisis management training should be mandatory for all residents at DRMC (%)	0	0	52.6	47.3
Team debriefing after a code blue or crisis scenario is important (%)	0	0	47.3	52.6
I would like to participate more in simulated crisis scenarios and mock codes and believe that they should be held more frequently (%)	0	0	47.3	52.6

**Table 3:** Survey questionnaire results after seven months DRMC: Desert Regional Medical Center

May 2019 (n = 24)	Strongly Disagree	Disagree	Agree	Strongly Agree
I am confident in my abilities and I am comfortable in running a code blue without an attending physician (%)	4.2	16.7	58.3	20.8
I have a clear understanding of my role during a code blue (%)	4.2	8.3	62.5	25
I feel comfortable announcing my role and communicating it with the resuscitation team during a crisis (%)	4.2	8.3	66.7	20.8
I feel comfortable assessing the effectiveness of chest compressions (%)	4.2	12.5	62.5	20.8
I feel comfortable drawing up resuscitation medications during a code blue scenario (%)	16.7	37.5	33.3	12.5
I feel comfortable operating the defibrillator during a code blue (%)	12.5	29.2	50	8.3
The use of mock code blue drills will play an important role in preparing me to handle crisis situations in the future (%)	0.0	0.0	70.8	29.2
Practicing multi-disciplinary, team-based mock code blue scenarios at DRMC would make me more comfortable in code blue situations in the future (%)	0.0	0.0	58.3	41.7
Practicing multi-disciplinary, team-based mock code blue scenarios will improve patient outcome (%)	0.0	0.0	50.0	50.0
Code blue and crisis management training should be mandatory for all residents at DRMC (%)	0.0	4.2	62.5	33.3
Team debriefing after a code blue or crisis scenario is important (%)	0.0	4.2	54.2	41.7
I would like to participate more in simulated crisis scenarios and mock codes and believe that they should be held more frequently (%)	0.0	4.2	58.3	37.5

## Discussion

At the conclusion of the seven-month study period involving the Desert Regional Medical Center internal medicine residency program, we noticed that there was an increase in self-reported improvement in the ability to lead a code blue scenario without an attending physician, understanding of roles of each code blue team member, comfort in communicating with team members, ability to assess the quality of chest compression, and operating code blue equipment such as a defibrillator or medications (Figures 1-6). There was an increase in perception among residents by the end of the study period that additional code blue drills will improve their comfort and ability to handle crisis situations in the future, as well as the likelihood that it would improve patient outcomes (Figures 7-9). Interestingly, we noticed that there was a decrease in those that strongly agreed and an increase in the percentage of residents that disagreed with the idea that code blue and crisis management training should be mandatory for all residents at DRMC (Figure 10). This deviation of thought maybe secondary to residents who, as they progress in their training, believe that it is not relevant to their chosen subspecialty.

Monthly data for the length of cardiac resuscitative efforts from October 2018 to April 2019 showed that a large majority of efforts lasted less than five minutes but no discernable pattern of change could be appreciated over the course of the study (Figure 13). With regards to metrics recorded for reason resuscitative efforts were stopped for patients with cardiac arrest, we find an increasing trend of death and decreasing trend of survival despite improvement in resident self-reported competency regarding crisis scenarios (Figure 14). These findings may be confounded based on the transient population demographics of Palm Springs, California, throughout the year. Affluent retirees who travel to warmer climates to avoid colder temperatures during the North American winters are commonly referred to as “snowbirds.” These snowbirds reside in Palm Springs and the surrounding Coachella Valley cities during the fall through winter months due to the warm local climate and leave during spring and summer months when temperatures rise in excess of 45° Celsius [5]. One possible conclusion could be made that the increased population density of snowbirds and presumed history of medical compliance may lead to better controlled and less severe medical comorbidities, increasing patient survivability after cardiac arrest. This has been suggested in a study by Smith et al., demonstrating that 63% of snowbirds rated their health as good or excellent, compared to 49% of "stayers" [6]. Additional studies would be needed to further investigate this. For patients who undergo cardiac arrest and were successfully resuscitated, we found that there was an increasing trend in the percentage of patients alive at the time of hospital discharge (Figure 15). Monthly increases in the percentage of patients who survive to discharge is likely secondary to monthly improvements in the resident’s ability to lead a code blue scenario and ability to assess the effectiveness of chest compressions to ensure adequate cerebral and cardiac perfusion performed by the code blue team.

There were several weaknesses in our study. The initial recruitment phase of the study only managed to successfully obtain 19 participants, whereas 24 was obtained at the end of the study. This potentially leads to an increased bias towards higher perceived competence at the end of the study period. The confidential and voluntary basis of subject recruitment made it difficult to ensure that the same subjects participated making data interpretation difficult. The time of the initial study date was also a weakness. A more accurate assessment of a resident's competency as a result of simulations could be made if the study started at the beginning of the residency academic year in July versus October.

## Conclusions

The utilization of simulated code blue scenarios and dedicated ACLS didactic lectures to supplement required BLS/ACLS certification for residents in training has demonstrated its ability to improve self-reported competency in crisis scenarios as well as a measurable reduction in patient mortality. We hope that the success of our study provides proof of concept for further investment of time and resources in simulated crisis scenarios for improved patient outcomes in other medical training programs.
